# Hexakis(1*H*-imidazole-κ*N* ^3^)cobalt(II) triaqua­tris(1*H*-imidazole-κ*N* ^3^)cobalt(II) bis­(naphthalene-1,4-dicarboxyl­ate)

**DOI:** 10.1107/S1600536809023794

**Published:** 2009-06-27

**Authors:** Jing-Jing Nie, Jun-Hua Li, Duan-Jun Xu

**Affiliations:** a Department of Chemistry, Zhejiang University, People’s Republic of China

## Abstract

The asymmetric unit of the title compound, [Co(C_3_H_4_N_2_)_6_][Co(C_3_H_4_N_2_)_3_(H_2_O)_3_](C_12_H_6_O_4_)_2_, contains two halves of crystallographically independent Co^II^ complex cations, each assuming a distorted octa­hedral geometry, and one uncoordinated naphthalene-1,4-dicarboxyl­ate dianion. One Co^II^ cation is located on an inversion center and is coordinated by six imidazole mol­ecules, while the other Co^II^ cation is located on a twofold rotation axis and is coordinated by three water and three imidazole mol­ecules. The uncoordinated naphthalene-1,4-dicarboxyl­ate dianion links both Co^II^ complex cations *via* O—H⋯O and N—H⋯O hydrogen bonding. One imidazole ligand is equally disordered over two sites about a twofold rotation axis, while the coordinated N atom of the imidazole is located on the twofold rotation axis. One water O atom has site symmetry 2.

## Related literature

For general background to the nature of π-π stacking, see: Su & Xu (2004 ▶); Xu *et al.* (2007 ▶). For related structures, see: Derissen *et al.* (1979 ▶); Li *et al.* (2008*a* ▶,*b* ▶).
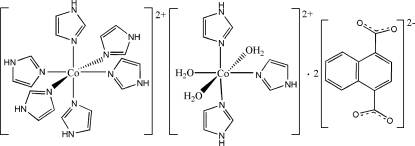


## Experimental

### 

#### Crystal data

[Co(C_3_H_4_N_2_)_6_][Co(C_3_H_4_N_2_)_3_(H_2_O)_3_](C_12_H_6_O_4_)_2_
*M* *_r_* = 1212.98
Orthorhombic, 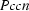

*a* = 29.388 (3) Å
*b* = 9.3275 (11) Å
*c* = 20.475 (2) Å
*V* = 5612.5 (10) Å^3^
*Z* = 4
Mo *K*α radiation
μ = 0.67 mm^−1^
*T* = 294 K
0.36 × 0.32 × 0.26 mm

#### Data collection

Rigaku R-AXIS RAPID IP diffractometer
Absorption correction: multi-scan (*ABSCOR*; Higashi, 1995 ▶) *T*_min_ = 0.735, *T*_max_ = 0.840
57832 measured reflections
5058 independent reflections
3916 reflections with *I* > 2σ(*I*)
*R*_int_ = 0.061

#### Refinement

*R*[*F* ^2^ > 2σ(*F* ^2^)] = 0.039
*wR*(*F* ^2^) = 0.100
*S* = 1.07
5058 reflections
367 parameters
5 restraints
H-atom parameters constrained
Δρ_max_ = 0.82 e Å^−3^
Δρ_min_ = −0.41 e Å^−3^

### 

Data collection: *PROCESS-AUTO* (Rigaku, 1998 ▶); cell refinement: *PROCESS-AUTO*; data reduction: *CrystalStructure* (Rigaku/MSC, 2002 ▶); program(s) used to solve structure: *SIR92* (Altomare *et al.*, 1993 ▶); program(s) used to refine structure: *SHELXL97* (Sheldrick, 2008 ▶); molecular graphics: *ORTEP-3 for Windows* (Farrugia, 1997 ▶); software used to prepare material for publication: *WinGX* (Farrugia, 1999 ▶).

## Supplementary Material

Crystal structure: contains datablocks I, global. DOI: 10.1107/S1600536809023794/hk2715sup1.cif

Structure factors: contains datablocks I. DOI: 10.1107/S1600536809023794/hk2715Isup2.hkl

Additional supplementary materials:  crystallographic information; 3D view; checkCIF report

## Figures and Tables

**Table 1:** Selected bond lengths (Å)

Co1—N1	2.146 (2)
Co1—N3	2.165 (2)
Co1—N5	2.174 (2)
Co2—O1*W*	2.1864 (17)
Co2—O2*W*	2.064 (2)
Co2—N7	2.166 (2)
Co2—N9	2.101 (3)

**Table 2:** Hydrogen-bond geometry (Å, °)

*D*—H⋯*A*	*D*—H	H⋯*A*	*D*⋯*A*	*D*—H⋯*A*
O1*W*—H1*A*⋯O4	0.93	1.85	2.768 (3)	168
O1*W*—H1*B*⋯O1^i^	0.85	2.04	2.883 (3)	173
O2*W*—H2*A*⋯O3	0.85	1.79	2.625 (3)	171
N2—H2*N*⋯O4	0.86	1.87	2.725 (3)	174
N4—H4*N*⋯O2^ii^	0.86	1.91	2.766 (3)	178
N6—H6*N*⋯O2^iii^	0.86	1.97	2.827 (3)	176
N8—H8*N*⋯O1^iv^	0.86	2.03	2.869 (3)	166
N10—H10*A*⋯O3^v^	0.86	1.89	2.658 (5)	149

Symmetry codes: (i) 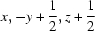; (ii) 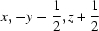; (iii) 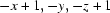; (iv) 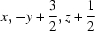; (v) 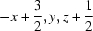.

**Fig. 1.**
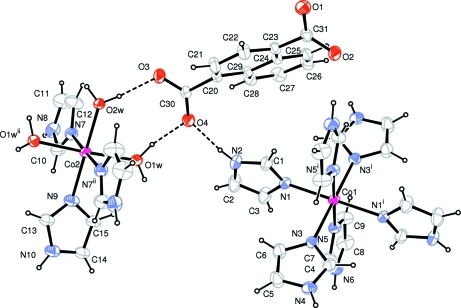
The molecular structure of the title compound with 30% probability displacement ellipsoids (arbitrary spheres for H atoms). One of the disordered imidazole components has been omitted for clarity. Dashed lines indicate hydrogen bonding [symmetry codes: (i) -x + 1, -y, -z + 1; (ii) -x + 3/2, -y + 3/2, z].

## supplementary crystallographic information

### Comment

As part of our ongoing investigation on the nature of π-π stacking (Su & Xu, 2004; Xu *et al.*, 2007), the title compound incorporating naphthalenedicarboxylate has recently been prepared in the laboratory and its crystal structure is reported here.

The asymmetric unit contains one uncoordinated naphthalenedicarboxylate dianion and two-halves of crystallographically independent Co^II^ complex cations. Both Co^II^ complexes assume distorted octahedral geometry. The Co1 atom is located on an inversion center and coordinated by six imidazole ligands, while the Co2 atom is located on a twofold rotation axis and coordinated by three water molecules and three imidazole ligands (Fig. 1). In the Co2-containing complex cation, the O2W and N9 atoms are located on the twofold rotation axis. The N9-imidazole ring is equally disordered over two sites about the twofold rotation axis, and the N9-imidazole ring is tilted with respect to the twofold axis by an angle of 12.2 (2)°, which is similar to 11.9 (5)° found in the Ni^II^ analogue (Li *et al.* 2008*b*) and 14.2 (3)° found in the Mn^II^ analogue (Li *et al.*, 2008*a*). The coordination bond distances (Table 1) are significantly shorter than those found in the Mn^II^ analogue but longer than those in the Ni^II^ analogue.

The uncoordinated naphthalenedicarboxylate dianion links with both Co^II^ complex cations *via* O—H···O and N—H···O hydrogen bonding (Fig. 1 and Table 2). Two carboxyl groups are twisted with respect to the naphthalene ring system by dihedral angles of 53.6 (3)° and 48.9 (3)°, which are larger than those found in the structure of free naphthalenedicarboxylic acid (*ca* 40°; Derissen *et al.*, 1979). No π-π stacking is observed between aromatic rings in the crystal structure.

### Experimental

A water-ethanol solution (20 ml, 1:2) of naphthalene-1,4-dicarboxyllic acid (0.11 g, 0.5 mmol) and sodium carbonate (0.053 g, 0.5 mmol) was refluxed for 0.5 h, then cobalt chloride hexahydrate (0.12 g, 0.5 mmol) was added to the above solution. The reaction mixture was refluxed for a further 4 h, then imidazole (0.10 g, 1.5 mmol) was added to the above solution and the reaction mixture was refluxed for another 0.5 h. After cooling to room temperature the solution was filtered. The single crystals of the title compound were obtained from the filtrate after one week.

### Refinement

The N9-containing imidazole is disordered over two sites about a twofold rotation axis, but the N9 atom is located on the twofold axis. The disordered components were refined with a half site occupancy. In the structure refinement, the coordinates of the N9 atom were refined by introducing an artificial bias of 0.02 (in fraction) to its *x* and *y* parameters, after several cycles of refinement the coordinates of the N9 atom shifted to the initial values of (3/4, 3/4, 0.64726). Bond distances of the disordered imidazole were restrained. Water H atoms were located in a difference Fourier map and refined as riding in as-found relative positions with *U*_iso_(H) = 1.5*U*_eq_(O). Other H atoms were placed in calculated positions with C—H = 0.93 Å and N—H = 0.86 Å, and refined in riding mode with *U*_iso_(H) = 1.2*U*_eq_(C,*N*).

### Crystal data

**Table:**

[Co(C_3_H_4_N_2_)_6_][Co(C_3_H_4_N_2_)_3_(H_2_O)_3_](C_12_H_6_O_4_)_2_	*F*(000) = 2512
*M**_r_* = 1212.98	*D*_x_ = 1.436 Mg m^−^^3^
Orthorhombic, *P**c**c**n*	Mo *K*α radiation, λ = 0.71073 Å
Hall symbol: -P 2ab 2ac	Cell parameters from 5022 reflections
*a* = 29.388 (3) Å	θ = 1.6–25.0°
*b* = 9.3275 (11) Å	µ = 0.67 mm^−^^1^
*c* = 20.475 (2) Å	*T* = 294 K
*V* = 5612.5 (10) Å^3^	Prism, pink
*Z* = 4	0.36 × 0.32 × 0.26 mm

### Data collection

**Table:**

Rigaku R-AXIS RAPID IP diffractometer	5058 independent reflections
Radiation source: fine-focus sealed tube	3916 reflections with *I* > 2σ(*I*)
graphite	*R*_int_ = 0.061
Detector resolution: 10.0 pixels mm^-1^	θ_max_ = 25.2°, θ_min_ = 1.4°
ω scans	*h* = −35→33
Absorption correction: multi-scan (*ABSCOR*; Higashi, 1995)	*k* = −11→11
*T*_min_ = 0.735, *T*_max_ = 0.840	*l* = −23→24
57832 measured reflections	

### Refinement

**Table:**

Refinement on *F*^2^	Primary atom site location: structure-invariant direct methods
Least-squares matrix: full	Secondary atom site location: difference Fourier map
*R*[*F*^2^ > 2σ(*F*^2^)] = 0.039	Hydrogen site location: inferred from neighbouring sites
*wR*(*F*^2^) = 0.100	H-atom parameters constrained
*S* = 1.07	*w* = 1/[σ^2^(*F*_o_^2^) + (0.0416*P*)^2^ + 3.4164*P*] where *P* = (*F*_o_^2^ + 2*F*_c_^2^)/3
5058 reflections	(Δ/σ)_max_ = 0.001
367 parameters	Δρ_max_ = 0.82 e Å^−^^3^
5 restraints	Δρ_min_ = −0.41 e Å^−^^3^

### Special details

**Table:**

Geometry. All e.s.d.'s (except the e.s.d. in the dihedral angle between two l.s. planes) are estimated using the full covariance matrix. The cell e.s.d.'s are taken into account individually in the estimation of e.s.d.'s in distances, angles and torsion angles; correlations between e.s.d.'s in cell parameters are only used when they are defined by crystal symmetry. An approximate (isotropic) treatment of cell e.s.d.'s is used for estimating e.s.d.'s involving l.s. planes.
Refinement. Refinement of *F*^2^ against ALL reflections. The weighted *R*-factor *wR* and goodness of fit *S* are based on *F*^2^, conventional *R*-factors *R* are based on *F*, with *F* set to zero for negative *F*^2^. The threshold expression of *F*^2^ > σ(*F*^2^) is used only for calculating *R*-factors(gt) *etc*. and is not relevant to the choice of reflections for refinement. *R*-factors based on *F*^2^ are statistically about twice as large as those based on *F*, and *R*- factors based on ALL data will be even larger.

### Fractional atomic coordinates and isotropic or equivalent isotropic displacement parameters (Å^2^)

**Table:**

	*x*	*y*	*z*	*U*_iso_*/*U*_eq_	Occ. (<1)
Co1	0.5000	0.0000	0.5000	0.03475 (13)	
Co2	0.7500	0.7500	0.54463 (2)	0.03692 (14)	
N1	0.53139 (7)	0.2076 (2)	0.50438 (10)	0.0415 (5)	
N2	0.58320 (9)	0.3736 (3)	0.49130 (13)	0.0592 (7)	
H2N	0.6056	0.4202	0.4747	0.071*	
N3	0.54621 (7)	−0.0709 (2)	0.57572 (10)	0.0408 (5)	
N4	0.58380 (8)	−0.2194 (2)	0.63945 (11)	0.0500 (6)	
H4N	0.5921	−0.2979	0.6580	0.060*	
N5	0.45060 (7)	0.0639 (2)	0.57347 (10)	0.0434 (5)	
N6	0.41451 (9)	0.0695 (3)	0.66759 (12)	0.0575 (6)	
H6N	0.4088	0.0574	0.7084	0.069*	
N7	0.72100 (8)	0.9634 (2)	0.54290 (11)	0.0467 (5)	
N8	0.69494 (9)	1.1752 (3)	0.57111 (14)	0.0601 (7)	
H8N	0.6866	1.2456	0.5955	0.072*	
O1	0.65890 (7)	0.0726 (2)	0.13247 (9)	0.0575 (5)	
O2	0.60816 (7)	−0.02645 (19)	0.19969 (9)	0.0536 (5)	
O3	0.69702 (9)	0.6180 (3)	0.35989 (10)	0.0910 (9)	
O4	0.65260 (7)	0.5131 (2)	0.43005 (9)	0.0614 (6)	
O1W	0.68251 (6)	0.65158 (18)	0.54162 (8)	0.0453 (4)	
H1A	0.6733	0.6170	0.5012	0.068*	
H1B	0.6772	0.5888	0.5708	0.068*	
O2W	0.7500	0.7500	0.44381 (11)	0.0510 (7)	
H2A	0.7342	0.6989	0.4182	0.077*	
C1	0.56663 (11)	0.2500 (3)	0.47050 (15)	0.0567 (8)	
H1	0.5787	0.1988	0.4356	0.068*	
C2	0.55829 (13)	0.4123 (3)	0.54329 (19)	0.0742 (10)	
H2	0.5624	0.4933	0.5691	0.089*	
C3	0.52607 (12)	0.3104 (3)	0.55061 (16)	0.0646 (9)	
H3	0.5036	0.3106	0.5826	0.078*	
C4	0.55044 (10)	−0.2038 (3)	0.59620 (13)	0.0495 (7)	
H4	0.5321	−0.2788	0.5820	0.059*	
C5	0.60213 (11)	−0.0882 (3)	0.64854 (17)	0.0675 (9)	
H5	0.6260	−0.0648	0.6765	0.081*	
C6	0.57913 (11)	0.0025 (3)	0.60917 (16)	0.0602 (8)	
H6	0.5848	0.1003	0.6053	0.072*	
C7	0.45256 (10)	0.0328 (3)	0.63626 (13)	0.0499 (7)	
H7	0.4775	−0.0098	0.6563	0.060*	
C8	0.38665 (12)	0.1295 (4)	0.62264 (17)	0.0712 (9)	
H8	0.3577	0.1665	0.6300	0.085*	
C9	0.40880 (11)	0.1255 (4)	0.56533 (16)	0.0639 (9)	
H9	0.3974	0.1595	0.5259	0.077*	
C10	0.71170 (11)	1.0496 (3)	0.59131 (16)	0.0599 (8)	
H10	0.7162	1.0263	0.6350	0.072*	
C11	0.69356 (13)	1.1707 (4)	0.50581 (19)	0.0776 (11)	
H11	0.6836	1.2429	0.4779	0.093*	
C12	0.70948 (13)	1.0405 (4)	0.48858 (17)	0.0750 (10)	
H12	0.7122	1.0078	0.4459	0.090*	
C20	0.65783 (9)	0.4146 (3)	0.32301 (12)	0.0404 (6)	
C21	0.69252 (10)	0.3443 (3)	0.29285 (14)	0.0558 (8)	
H21	0.7224	0.3683	0.3032	0.067*	
C22	0.68432 (10)	0.2362 (3)	0.24652 (14)	0.0535 (7)	
H22	0.7088	0.1905	0.2267	0.064*	
C23	0.64097 (9)	0.1970 (3)	0.23010 (12)	0.0391 (6)	
C24	0.60349 (8)	0.2713 (2)	0.25851 (11)	0.0358 (6)	
C25	0.55755 (9)	0.2425 (3)	0.24077 (13)	0.0446 (6)	
H25	0.5514	0.1701	0.2108	0.054*	
C26	0.52253 (10)	0.3182 (3)	0.26665 (14)	0.0539 (7)	
H26	0.4928	0.2956	0.2551	0.065*	
C27	0.53077 (10)	0.4302 (3)	0.31069 (14)	0.0559 (8)	
H27	0.5066	0.4831	0.3272	0.067*	
C28	0.57421 (10)	0.4620 (3)	0.32946 (13)	0.0463 (7)	
H28	0.5792	0.5366	0.3587	0.056*	
C29	0.61191 (8)	0.3829 (2)	0.30492 (11)	0.0368 (6)	
C30	0.66975 (10)	0.5234 (3)	0.37521 (13)	0.0467 (7)	
C31	0.63550 (9)	0.0723 (3)	0.18330 (13)	0.0425 (6)	
N9	0.7500	0.7500	0.64726 (14)	0.0532 (6)	
N10	0.76923 (15)	0.7145 (5)	0.74775 (19)	0.0532 (6)	0.50
H10A	0.7858	0.7124	0.7823	0.064*	0.50
C13	0.78454 (12)	0.7510 (6)	0.68841 (17)	0.0532 (6)	0.50
H13	0.8145	0.7733	0.6780	0.064*	0.50
C14	0.72418 (16)	0.6813 (6)	0.7466 (2)	0.0532 (6)	0.50
H14	0.7056	0.6512	0.7806	0.064*	0.50
C15	0.71334 (12)	0.7034 (6)	0.68278 (19)	0.0532 (6)	0.50
H15	0.6845	0.6885	0.6653	0.064*	0.50

### Atomic displacement parameters (Å^2^)

**Table:**

	*U*^11^	*U*^22^	*U*^33^	*U*^12^	*U*^13^	*U*^23^
Co1	0.0411 (3)	0.0299 (2)	0.0333 (2)	0.0009 (2)	0.0015 (2)	0.00306 (19)
Co2	0.0484 (3)	0.0339 (2)	0.0285 (2)	−0.0068 (2)	0.000	0.000
N1	0.0466 (13)	0.0354 (11)	0.0424 (12)	−0.0041 (10)	−0.0008 (10)	0.0050 (9)
N2	0.0621 (16)	0.0473 (14)	0.0682 (17)	−0.0199 (12)	−0.0011 (13)	0.0039 (12)
N3	0.0471 (13)	0.0333 (11)	0.0422 (12)	0.0041 (10)	−0.0022 (10)	0.0034 (9)
N4	0.0532 (14)	0.0460 (13)	0.0508 (14)	0.0101 (11)	−0.0043 (11)	0.0111 (11)
N5	0.0484 (14)	0.0390 (11)	0.0427 (13)	0.0013 (10)	0.0074 (10)	0.0017 (10)
N6	0.0685 (17)	0.0586 (15)	0.0454 (14)	−0.0035 (13)	0.0166 (13)	−0.0064 (12)
N7	0.0523 (14)	0.0396 (12)	0.0482 (13)	−0.0011 (10)	−0.0006 (11)	0.0016 (10)
N8	0.0638 (17)	0.0409 (13)	0.0755 (18)	0.0044 (12)	0.0103 (14)	−0.0018 (13)
O1	0.0802 (15)	0.0484 (11)	0.0438 (11)	−0.0076 (10)	0.0182 (10)	−0.0122 (9)
O2	0.0706 (14)	0.0447 (11)	0.0454 (11)	−0.0173 (10)	0.0079 (10)	−0.0124 (9)
O3	0.127 (2)	0.1031 (18)	0.0424 (12)	−0.0803 (17)	0.0085 (13)	−0.0151 (12)
O4	0.0803 (15)	0.0672 (13)	0.0368 (11)	−0.0317 (11)	0.0084 (10)	−0.0135 (9)
O1W	0.0565 (11)	0.0440 (10)	0.0355 (9)	−0.0124 (8)	0.0019 (8)	0.0015 (8)
O2W	0.0704 (18)	0.0545 (15)	0.0282 (13)	−0.0297 (14)	0.000	0.000
C1	0.067 (2)	0.0487 (16)	0.0548 (18)	−0.0153 (15)	0.0080 (15)	−0.0052 (14)
C2	0.086 (3)	0.0440 (18)	0.093 (3)	−0.0116 (17)	0.006 (2)	−0.0203 (17)
C3	0.071 (2)	0.0473 (16)	0.075 (2)	−0.0077 (16)	0.0161 (17)	−0.0168 (16)
C4	0.0554 (18)	0.0394 (14)	0.0536 (17)	−0.0004 (12)	−0.0081 (14)	0.0074 (12)
C5	0.069 (2)	0.0526 (18)	0.081 (2)	0.0016 (16)	−0.0325 (18)	0.0018 (17)
C6	0.064 (2)	0.0392 (15)	0.078 (2)	0.0010 (14)	−0.0231 (17)	0.0040 (15)
C7	0.0530 (17)	0.0553 (16)	0.0414 (16)	−0.0005 (13)	0.0080 (13)	−0.0039 (13)
C8	0.061 (2)	0.078 (2)	0.074 (2)	0.0181 (18)	0.0243 (19)	0.0008 (19)
C9	0.061 (2)	0.072 (2)	0.0585 (19)	0.0217 (17)	0.0084 (15)	0.0112 (16)
C10	0.083 (2)	0.0414 (15)	0.0557 (19)	0.0021 (15)	0.0067 (16)	0.0006 (14)
C11	0.093 (3)	0.060 (2)	0.080 (3)	0.0272 (19)	−0.006 (2)	0.0116 (18)
C12	0.104 (3)	0.064 (2)	0.057 (2)	0.025 (2)	−0.0131 (19)	0.0038 (16)
C20	0.0455 (16)	0.0407 (14)	0.0350 (13)	−0.0095 (12)	0.0003 (11)	−0.0058 (11)
C21	0.0394 (16)	0.070 (2)	0.0583 (18)	−0.0134 (14)	−0.0011 (14)	−0.0199 (15)
C22	0.0403 (16)	0.0631 (18)	0.0572 (18)	−0.0031 (14)	0.0063 (13)	−0.0204 (15)
C23	0.0419 (15)	0.0399 (13)	0.0354 (13)	−0.0045 (11)	0.0004 (11)	−0.0069 (11)
C24	0.0392 (14)	0.0375 (13)	0.0306 (12)	−0.0033 (11)	−0.0030 (10)	0.0008 (10)
C25	0.0427 (16)	0.0503 (15)	0.0409 (14)	−0.0045 (13)	−0.0054 (12)	−0.0050 (12)
C26	0.0368 (16)	0.0713 (19)	0.0536 (17)	0.0014 (14)	−0.0097 (13)	0.0009 (16)
C27	0.0486 (18)	0.0635 (19)	0.0557 (18)	0.0180 (15)	−0.0016 (14)	−0.0019 (15)
C28	0.0566 (18)	0.0408 (14)	0.0416 (15)	0.0073 (13)	−0.0019 (13)	−0.0067 (12)
C29	0.0443 (15)	0.0342 (12)	0.0318 (13)	−0.0026 (11)	−0.0011 (11)	−0.0024 (10)
C30	0.0586 (18)	0.0444 (15)	0.0373 (15)	−0.0163 (13)	−0.0036 (13)	−0.0058 (12)
C31	0.0500 (16)	0.0382 (14)	0.0394 (15)	−0.0008 (12)	−0.0014 (12)	−0.0079 (11)
N9	0.0688 (13)	0.0549 (14)	0.0359 (9)	0.0052 (12)	0.000	0.000
N10	0.0688 (13)	0.0549 (14)	0.0359 (9)	0.0052 (12)	0.000	0.000
C13	0.0688 (13)	0.0549 (14)	0.0359 (9)	0.0052 (12)	0.000	0.000
C14	0.0688 (13)	0.0549 (14)	0.0359 (9)	0.0052 (12)	0.000	0.000
C15	0.0688 (13)	0.0549 (14)	0.0359 (9)	0.0052 (12)	0.000	0.000

### Geometric parameters (Å, °)

**Table:**

Co1—N1	2.146 (2)	C4—H4	0.9300
Co1—N1^i^	2.146 (2)	C5—C6	1.350 (4)
Co1—N3	2.165 (2)	C5—H5	0.9300
Co1—N3^i^	2.165 (2)	C6—H6	0.9300
Co1—N5	2.174 (2)	C7—H7	0.9300
Co1—N5^i^	2.174 (2)	C8—C9	1.342 (4)
Co2—O1W^ii^	2.1864 (17)	C8—H8	0.9300
Co2—O1W	2.1864 (17)	C9—H9	0.9300
Co2—O2W	2.064 (2)	C10—H10	0.9300
Co2—N7^ii^	2.166 (2)	C11—C12	1.348 (5)
Co2—N7	2.166 (2)	C11—H11	0.9300
Co2—N9	2.101 (3)	C12—H12	0.9300
N1—C1	1.308 (3)	C20—C21	1.361 (4)
N1—C3	1.356 (4)	C20—C29	1.430 (3)
N2—C1	1.322 (4)	C20—C30	1.515 (3)
N2—C2	1.341 (4)	C21—C22	1.405 (4)
N2—H2N	0.8600	C21—H21	0.9300
N3—C4	1.314 (3)	C22—C23	1.368 (4)
N3—C6	1.369 (4)	C22—H22	0.9300
N4—C4	1.329 (3)	C23—C24	1.426 (3)
N4—C5	1.350 (4)	C23—C31	1.515 (3)
N4—H4N	0.8600	C24—C25	1.424 (4)
N5—C7	1.319 (3)	C24—C29	1.431 (3)
N5—C9	1.366 (4)	C25—C26	1.356 (4)
N6—C7	1.334 (4)	C25—H25	0.9300
N6—C8	1.353 (4)	C26—C27	1.401 (4)
N6—H6N	0.8600	C26—H26	0.9300
N7—C10	1.305 (4)	C27—C28	1.366 (4)
N7—C12	1.367 (4)	C27—H27	0.9300
N8—C10	1.336 (4)	C28—C29	1.423 (4)
N8—C11	1.338 (4)	C28—H28	0.9300
N8—H8N	0.8600	N9—C13	1.3190 (10)
O1—C31	1.248 (3)	N9—C13^ii^	1.3190 (10)
O2—C31	1.268 (3)	N9—C15^ii^	1.3706 (10)
O3—C30	1.232 (3)	N9—C15	1.3706 (10)
O4—C30	1.234 (3)	N10—C13	1.3395 (10)
O1W—H1A	0.9287	N10—C14	1.3598 (10)
O1W—H1B	0.8504	N10—H10A	0.8600
O2W—H2A	0.8475	C13—H13	0.9300
C1—H1	0.9300	C14—C15	1.3597 (10)
C2—C3	1.350 (4)	C14—H14	0.9300
C2—H2	0.9300	C15—H15	0.9300
C3—H3	0.9300		
			
N1—Co1—N1^i^	180.00 (10)	N5—C7—N6	112.0 (3)
N1—Co1—N3	88.64 (8)	N5—C7—H7	124.0
N1^i^—Co1—N3	91.36 (8)	N6—C7—H7	124.0
N1—Co1—N3^i^	91.36 (8)	C9—C8—N6	106.8 (3)
N1^i^—Co1—N3^i^	88.64 (8)	C9—C8—H8	126.6
N3—Co1—N3^i^	180.00 (12)	N6—C8—H8	126.6
N1—Co1—N5	90.61 (8)	C8—C9—N5	110.0 (3)
N1^i^—Co1—N5	89.39 (8)	C8—C9—H9	125.0
N3—Co1—N5	90.41 (8)	N5—C9—H9	125.0
N3^i^—Co1—N5	89.59 (8)	N7—C10—N8	112.5 (3)
N1—Co1—N5^i^	89.38 (8)	N7—C10—H10	123.8
N1^i^—Co1—N5^i^	90.62 (8)	N8—C10—H10	123.8
N3—Co1—N5^i^	89.59 (8)	N8—C11—C12	106.2 (3)
N3^i^—Co1—N5^i^	90.41 (8)	N8—C11—H11	126.9
N5—Co1—N5^i^	180.00 (8)	C12—C11—H11	126.9
O2W—Co2—N9	180.000 (1)	C11—C12—N7	110.3 (3)
O2W—Co2—N7^ii^	89.06 (6)	C11—C12—H12	124.9
N9—Co2—N7^ii^	90.94 (6)	N7—C12—H12	124.8
O2W—Co2—N7	89.06 (6)	C21—C20—C29	119.3 (2)
N9—Co2—N7	90.94 (6)	C21—C20—C30	118.0 (2)
N7^ii^—Co2—N7	178.12 (12)	C29—C20—C30	122.7 (2)
O2W—Co2—O1W^ii^	88.38 (4)	C20—C21—C22	121.6 (3)
N9—Co2—O1W^ii^	91.62 (4)	C20—C21—H21	119.2
N7^ii^—Co2—O1W^ii^	91.63 (8)	C22—C21—H21	119.2
N7—Co2—O1W^ii^	88.32 (8)	C23—C22—C21	121.2 (3)
O2W—Co2—O1W	88.38 (4)	C23—C22—H22	119.4
N9—Co2—O1W	91.62 (4)	C21—C22—H22	119.4
N7^ii^—Co2—O1W	88.32 (8)	C22—C23—C24	119.3 (2)
N7—Co2—O1W	91.63 (8)	C22—C23—C31	117.4 (2)
O1W^ii^—Co2—O1W	176.77 (9)	C24—C23—C31	123.3 (2)
C1—N1—C3	104.3 (2)	C25—C24—C23	122.4 (2)
C1—N1—Co1	126.23 (19)	C25—C24—C29	118.1 (2)
C3—N1—Co1	128.12 (19)	C23—C24—C29	119.4 (2)
C1—N2—C2	106.8 (3)	C26—C25—C24	121.4 (2)
C1—N2—H2N	126.6	C26—C25—H25	119.3
C2—N2—H2N	126.6	C24—C25—H25	119.3
C4—N3—C6	104.2 (2)	C25—C26—C27	120.6 (3)
C4—N3—Co1	125.18 (18)	C25—C26—H26	119.7
C6—N3—Co1	130.43 (17)	C27—C26—H26	119.7
C4—N4—C5	106.7 (2)	C28—C27—C26	120.3 (3)
C4—N4—H4N	126.7	C28—C27—H27	119.8
C5—N4—H4N	126.7	C26—C27—H27	119.8
C7—N5—C9	104.5 (2)	C27—C28—C29	121.0 (2)
C7—N5—Co1	125.79 (19)	C27—C28—H28	119.5
C9—N5—Co1	129.15 (19)	C29—C28—H28	119.5
C7—N6—C8	106.6 (3)	C28—C29—C20	122.4 (2)
C7—N6—H6N	126.7	C28—C29—C24	118.5 (2)
C8—N6—H6N	126.7	C20—C29—C24	119.1 (2)
C10—N7—C12	104.0 (3)	O3—C30—O4	123.6 (2)
C10—N7—Co2	129.5 (2)	O3—C30—C20	116.8 (2)
C12—N7—Co2	126.5 (2)	O4—C30—C20	119.7 (2)
C10—N8—C11	107.0 (3)	O1—C31—O2	124.8 (2)
C10—N8—H8N	126.5	O1—C31—C23	117.9 (2)
C11—N8—H8N	126.5	O2—C31—C23	117.2 (2)
Co2—O1W—H1A	115.7	C13—N9—C13^ii^	100.6 (4)
Co2—O1W—H1B	115.8	C13^ii^—N9—C15^ii^	105.6 (3)
H1A—O1W—H1B	109.5	C13—N9—C15	105.6 (3)
Co2—O2W—H2A	128.3	C15^ii^—N9—C15	115.9 (5)
N1—C1—N2	112.6 (3)	C13—N9—Co2	129.7 (2)
N1—C1—H1	123.7	C13^ii^—N9—Co2	129.7 (2)
N2—C1—H1	123.7	C15^ii^—N9—Co2	122.0 (2)
N2—C2—C3	106.3 (3)	C15—N9—Co2	122.0 (2)
N2—C2—H2	126.8	C13—N10—C14	111.6 (4)
C3—C2—H2	126.8	C13—N10—H10A	124.2
C2—C3—N1	109.9 (3)	C14—N10—H10A	124.2
C2—C3—H3	125.1	N9—C13—N10	108.6 (4)
N1—C3—H3	125.1	N9—C13—H13	125.7
N3—C4—N4	112.7 (3)	N10—C13—H13	125.7
N3—C4—H4	123.7	C15—C14—N10	102.2 (4)
N4—C4—H4	123.7	C15—C14—C14^ii^	94.7 (3)
C6—C5—N4	106.6 (3)	C15—C14—H14	128.9
C6—C5—H5	126.7	N10—C14—H14	128.9
N4—C5—H5	126.7	C14^ii^—C14—H14	130.1
C5—C6—N3	109.8 (3)	C14—C15—N9	111.9 (4)
C5—C6—H6	125.1	C14—C15—H15	124.0
N3—C6—H6	125.1	N9—C15—H15	124.0

Symmetry codes: (i) −*x*+1, −*y*, −*z*+1; (ii) −*x*+3/2, −*y*+3/2, *z*.

### Hydrogen-bond geometry (Å, °)

**Table:**

*D*—H···*A*	*D*—H	H···*A*	*D*···*A*	*D*—H···*A*
O1W—H1A···O4	0.93	1.85	2.768 (3)	168
O1W—H1B···O1^iii^	0.85	2.04	2.883 (3)	173
O2W—H2A···O3	0.85	1.79	2.625 (3)	171
N2—H2N···O4	0.86	1.87	2.725 (3)	174
N4—H4N···O2^iv^	0.86	1.91	2.766 (3)	178
N6—H6N···O2^i^	0.86	1.97	2.827 (3)	176
N8—H8N···O1^v^	0.86	2.03	2.869 (3)	166
N10—H10A···O3^vi^	0.86	1.89	2.658 (5)	149

Symmetry codes: (iii) *x*, −*y*+1/2, *z*+1/2; (iv) *x*, −*y*−1/2, *z*+1/2; (i) −*x*+1, −*y*, −*z*+1; (v) *x*, −*y*+3/2, *z*+1/2; (vi) −*x*+3/2, *y*, *z*+1/2.
